# Screening and overdiagnosis: public health implications

**DOI:** 10.1186/s40985-015-0012-1

**Published:** 2015-11-05

**Authors:** Jean-Luc Bulliard, Arnaud Chiolero

**Affiliations:** 1 Division of Chronic Diseases, Institute of social and preventive medicine (IUMSP), Lausanne University Hospital, Lausanne, Switzerland; 2 Observatoire valaisan de la santé (OVS), Sion, Switzerland

**Keywords:** Screening, Overdiagnosis, Overtreatment, Chronic diseases

## Abstract

Overdiagnosis is the diagnosis of an abnormality that bears no substantial health hazard and no benefit for patients to be aware of. Resulting mainly from the use of increasingly sensitive screening and diagnostic tests, as well as broadened definitions of conditions requiring an intervention, overdiagnosis is a growing but still largely misunderstood public health issue. Fear of missing a diagnosis or of litigation, financial incentives or patient’s need of reassurance are further causes of overdiagnosis. The main consequence of overdiagnosis is overtreatment. Treating an overdiagnosed condition bears no benefit but can cause harms and generates costs. Overtreatment also diverts health professionals from caring for those most severely ill. Recognition of overdiagnosis due to screening is challenging since it is rarely identifiable at the individual level and difficult to quantify precisely at the population level. Overdiagnosis exists even for screening of proven efficacy and efficiency. Measures to reduce overdiagnosis due to screening include heightened sensitization of health professionals and patients, active surveillance and deferred treatment until early signs of disease progression and prognosis estimation through biomarkers (including molecular) profiling. Targeted screening and balanced information on its risk and benefits would also help limit overdiagnosis. Research is needed to assess the public health burden and implications of overdiagnosis due to screening activity.

## Background

### What is overdiagnosis?

Overdiagnosis was originally defined as the diagnosis of an inconsequential disease (or *pseudodisease*), based on its clinicopathological characteristics, irrespective of its host [1]. It was *per se* unrelated to life expectancy or mode of detection. Advances in, and increasing use of, diagnostic and screening technologies, in a context where chronic diseases prevail, has led to the early detection of more diseases during their preclinical phase, i.e., before any symptom arises. This brought an epidemiological concept of overdiagnosis: the diagnosis of a condition that would have remained indolent in the patient’s lifetime if left undetected.

**Fig. 1:**
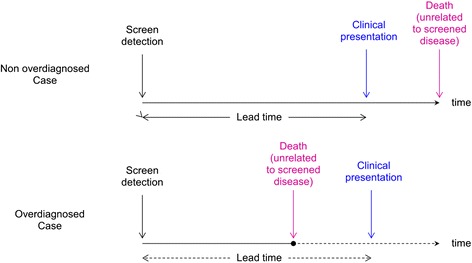
Overdiagnosis and lead time in screening (adapted from [2])

This concept ties overdiagnosis to screening and absence of gain in longevity. As such, any patient screen-detected with a cancer or a precancerous lesion who eventually dies from competing mortality will be considered as an overdiagnosed case if (and only if) death occurs before clinical manifestation of this cancer (Fig. 1). Overdiagnosis is not limited to cancer and can occur with any other screened-detected conditions, e.g., hypertension, diabetes, or osteoporosis. With this screening-related concept, the earlier detection occurs before clinical presentation of a disease (lead time), the greater the potential for overdiagnosis [2]. As overdiagnosis is a growing but still largely misunderstood public health issue, we address hereafter the main causes and consequences of overdiagnosis due to screening, and suggest ways to reduce this undesirable screening effect.

### What causes overdiagnosis?

Overdiagnosis has multiple causes (Table 1). The use of increasingly sensitive diagnostic tests is a major source of overdiagnosis [3]. These tests lead to findings, incidentally or not, of abnormalities of uncertain clinical hazard. Detection of non-life-threatening prostate cancer by determination of prostate-specific antigen (PSA), of small papillary thyroid cancer by ultrasonography, or of nonhypersecreting, benign adrenocortical adenomas (so-called incidentaloma) by computed tomography (CT) are some classical examples [4–6]. Insufficient knowledge of the natural history of the screened disease, a well-known pre-requisite to ensure the relevance of screening, also contributes to overdiagnosis [7, 8]. For instance, growing evidence supports that different forms of cancers are of variable progression rate, including indolent, regressive, slowly progressive and aggressive tumours. This is at odds with the implicit assumption of a unique, orderly and gradual progression of cancers on which screening is based [9]. Overdiagnosis can also result from the detection of precancerous lesions even with screening methods of established success, such as cervical intraepithelial neoplasia or polyp for cervical and colorectal cancer.

**Table 1:** Causes of overdiagnosis [14, 27]

• Screening and increasing sensitivity of diagnostic tests
• Incidental findings following screening or diagnostic tests
• Widening diagnosis criteria
• Confusion between risk and disease
• Physician’s fear of missing a disease or their patients expectations
• Financial incentives by pharmaceutical and diagnostic test industries
• Insufficient knowledge of the natural history of the disease

A more subtle form of overdiagnosis is due to the tendency to broaden the definition of conditions requiring treatment. This occurs for instance when screening targets a risk factor for a disease blurring the boundary between health and disease [10]. For instance, lowering the thresholds of blood pressure to define hypertension or pre-hypertension increases the number of patients diagnosed with a condition requiring follow-up and treatment. However, below a certain level of the risk factors, the associated absolute risk of disease is small and treatment bears little, if any, benefit [11]. This is due to the loglinear relationship between risk factors such as blood pressure and risk of associated diseases, which depends essentially on age for cardiovascular diseases [12]. Hence, for an identical reduction in blood pressure, the resulting absolute risk of disease reduction will be much smaller for individuals with blood pressure below a certain level compared with individuals with high level of blood pressure [13]. This leads to overtreatment. Following the same logic, diabetes, pre-diabetes, or gestational diabetes as well as osteoporosis can also be overdiagnosed. Use of the relative rather than the absolute risk magnifies the risk of these conditions and gives an overoptimistic impression of the degree of risk reduction that could be expected from screening and treating these conditions [11]. Fear of missing a diagnosis or of litigation, financial incentives or patient’s perceived need of reassurance are other causes of overdiagnosis [14] (Table 1).

### What are the consequences of overdiagnosis?

The main – and worst – consequence of overdiagnosis is overtreatment. By definition, treating an indolent disease bears no benefit but can cause harms, generates costs and wastes resources. This is particularly important for screening which, unlike other medical interventions, targets healthy subjects, already exposed to other side effects of screening, such as false-positive results [15, 16]. Notwithstanding, overtreatment shifts pervasively the attention and utilization of some care away from those most severely ill. Another under-recognised consequence of overdiagnosis is the psychological suffering due to the unnecessary labelling of people with a lifelong diagnosis [17].

### How to reduce overdiagnosis?

Once an abnormality is detected or a patient exceeds a set level of a risk factor, it is virtually impossible and often unethical not to investigate and treat the patient, even if the probability of overdiagnosis is high. This major challenge hinders the identifiability of overdiagnosis at the individual level, which, in turn, hampers its recognition by clinicians and patients. However, potential measures to reduce overdiagnosis exist and depend on the causes of overdiagnosis.

The fundamental precept *primum non nocere* must prevail against the broad “more is better” attitudes and nearly blind beliefs in new, more diagnostically sensitive technologies. Physicians have limited training and suboptimal ability to adequately assess screening benefit and overdiagnosis [18]. They should be provided with absolute measures of the risk reduction expected from screening, framed in an easy to understand manner, and aimed at sharing the screening decision with their patients [19]. Increasing awareness of health professionals and patients about overdiagnosis should contribute to fight against pressure to prescribe popular but inefficient screening tests and, to some extent, against financial incentives from diagnostic and pharmaceutical industries to perform them [20, 21].

As long as indolent lesions cannot be distinguished from aggressive ones at diagnosis, screening will unavoidably result in some overdiagnosis [16]. Active surveillance, deferred treatment until early signs of disease progression, and prognosis estimation through biomarkers (including molecular) profiling are among our best prospects to prevent overtreatment and to increase the benefit of screening [9, 22]. For instance, application of a clinically validated genomic assay has enabled an individual risk stratification of ductal carcinoma in situ (DCIS) of the breast and increased the number of patients benefiting from conserving surgery [23]. Screening should also be limited to tests/examinations and subpopulations for which the evidence-based benefit is strong, and abide to the recommended interscreening interval. Targeted screening and balanced information on its risk and benefits should help limit overdiagnosis. Comparative studies of screening strategies taking into account specific biomarkers and other patients’ characteristics are needed for different diseases. While “personalized” screening allows a better risk stratification of the patient and hence potentially prevent overtreatment, it could generate overdiagnosis, notably through incidental findings, e.g., following genome-wide sequencing [24].

It is essential to acknowledge that the balance between benefits, in terms of life saved, and overdiagnosis, can be highly challenging to quantify, particularly for cancer screening, and contains a substantial level of uncertainty [2, 25]. Further, the perception of this balance remains highly subjective, even for efficacious and cost-effective screening interventions. For instance, some (patients, physicians, scientific experts, public health authorities) may deem that 3 breast cancers overdiagnosed per life saved are well worth recommending regular mammography screening for women aged 50 to 70 years, whereas others may feel unconvinced by this balance [26].

## Conclusions

While increasingly sensitive screening and diagnostic tests enable to detect many potentially severe and socially relevant frequent chronic diseases at the earliest stages, they concomitantly expand the disease reservoir of subclinical conditions from which overdiagnosis occurs (Table 2). There is an urge to give a higher emphasis on the specificity of screening tests, particularly for universal and population-based screening, to sensitize the general public and health professionals that overdiagnosis occurs more frequently with screening, and to inform them about the potential implications of overdiagnosis. Research is also needed to assess the public health burden and implications of overdiagnosis due to screening activity.

**Table 2:** Key messages

• Overdiagnosis is the diagnosis of a condition that is not associated with a substantial health hazard; it causes overtreatment.
• Screening is a major cause of overdiagnosis.
• Patients and physicians should be informed about risk of overdiagnosis associated with screening. Targeted screening, active surveillance, and prognosis estimation allow prevention of overdiagnosis and of overtreatment.
• Research is needed to assess the public health burden of overdiagnosis due to screening activity.
